# A cross-cultural study of high-altitude botanical resources among diverse ethnic groups in Kashmir Himalaya, India

**DOI:** 10.1186/s13002-023-00582-8

**Published:** 2023-04-13

**Authors:** Shiekh Marifatul Haq, Muhammad Waheed, Aadil Abdullah Khoja, Muhammad Shoaib Amjad, Rainer W. Bussmann, Kishwar Ali

**Affiliations:** 1 Department of Ethnobotany, Institute of Botany, Ilia State University, Tbilisi, Georgia; 2 Department of Botany, University of Okara, Okara, 56300 Pakistan; 3 Department of Botany, Glocal University, Saharanpur, U.P 247121 India; 4 Department of Botany, Women University of Azad Jammu & Kashmir, Bagh, 12500 Pakistan; 5 Birmingham Institute of Forest Research, University of Birmingham, Birmingham, B15 2TT UK; 6 Department of Botany, Institute of Life Sciences, State Museum of Natural History, Karlsruhe, Germany; 7 College of General Education, University of Doha for Science and Technology, Al Tarafa, Jelaiah Street, Duhail North, P.O Box 24449, Doha, Qatar

**Keywords:** Cultural relationships, Linear regression model, Indicator values, Ethnic groups, Western Himalayas

## Abstract

**Background:**

In the Himalayas, traditional knowledge and biodiversity are strongly linked due to the symbiotic interaction between plant and cultural diversity, as well as the support provided by cultural memories, ecological awareness, and social norms. Our study was focused on documenting the vanishing knowledge in the Kashmir Himalaya with the following main objectives: 1) to document the ethnomedical and cultural knowledge of the local flora, 2) to evaluate the cross-cultural use of the flora in the region, and, finally, 3) to identify the key indicator species utilized by each ethnic group using multivariate statistical analysis.

**Methods:**

We used semi-structured questionnaires to conduct interviews with people of different ethnicity, gender, age, and occupational categories. The intercultural relationships of species utilization among ethnic groups were examined using a Venn diagram. The overall trends between the indicator values and the plant species used by diverse ethnic groups were illustrated using the linear regression model.

**Results:**

We recorded 46 species belonging to 25 different families used by the local people of the Kashmir Valley belonging to four ethnic groups (Gujjar, Bakarwal, Pahari, and Kashmiri). The dominant families recorded were Asteraceae and Ranunculaceae followed by Caprifoliaceae. Rhizomes were the most utilized plant part, followed by leaves. A total of 33 ailments were treated with plants, and gastrointestinal disorders were treated with most species followed by musculoskeletal diseases and dermatological problems. Across cultural relationships, the Gujjar and Pahari showed greater similarity (17%). This may be due to the fact that both ethnic groups share a common geographical landscape and are exogamous to each other. We identified key indicator species used by different ethnic groups with significant (*p* ≤ 0.05) values. For instance, in the Gujjar ethnic group, *Aconitum heterophyllum* and *Phytolacca acinosa* had significant indicator value, which was due to the fact that these plants were easily accessible and also had a wide range of uses. In contrast, the Bakarwal ethnic group showed different indicator species, with *Rheum spiciforme* and *Rhododendron campanulatum* being highly significant (*p* ≤ 0.05), because this ethnic group spends the majority of their time in high-altitude pastures, using a particularly wide variety of plant species for medicine, food, and fuelwood. While indicator values and plant usage were positively correlated for the Gujjar, Kashmiri, and Pahari ethnic groups, they were negatively correlated for the Bakarwal. The positive correlation indicates cultural preferences for certain plant use and underlines the cultural significance of each species. The current study reported new uses for the following species: raw roots of *Jurinea dolomiaea* used for tooth cleaning, seeds of *Verbascum thapsus* applied for respiratory diseases, and flowers of *Saussurea simpsoniana* given to anyone as a good luck wish.

**Conclusion:**

The current study highlights historical ethnic group stratifications and cultural standing while comparing reported taxa across cultures. Each ethnic group made extensive ethnomedical use of plants, and knowledge, originally transmitted verbally, is now available in writing for reference. This could pave the way for providing incentives to local communities to showcase their talents, celebrate them, and gain from potential development initiatives.

## Introduction

Local and indigenous communities often have a close link with the environment [1] and often living in natural habitats they hold tremendous traditional knowledge about the use of biotic resources [2]. It is well acknowledged that traditional medicine has contributed to the discovery of a wide variety of allopathic drugs [3, 4]. Forest products such as food, fodder, and medicine are an important livelihood source for communities [5]. Knowledge of medicinal plants and their usage are part of culture and knowledge institutions [6]. Local knowledge incorporates different elements including human cognition, social networks, cultural beliefs, local categorization systems, language, religion, and information access [7, 8].

Ethnobotanical documentation can contribute to the conservation of local plant diversity, culture, and their interactions [9, 10]. The identification of new ingredients for allopathic medications and formulations might be based on ethnobotanical research that records traditional knowledge [11, 12]. Because of the symbiotic relationship between plant diversity and cultures, and religious traditions and rituals, cultural diversity and biodiversity are closely related in the Himalayas [13, 14]. If appropriately channeled, plant resources can also give direct economic benefits through trade and play an important role in improving livelihoods [15], helping to alleviate issues such as unemployment and food insecurity.

Traditional medicines are important in many countries, e.g., China, India, and Japan [16]. In India, around 65% of the population particularly in rural areas is still dependent on traditional medicine for primary health care [17]. The most important health issues are often intestinal problems linked to inadequate sewage infrastructure and a lack of safe drinking water [18, 19]. The Himalayan region holds more than half of India's biodiversity [20], serving as a prime resource for food and medicine [8, 21]. Kashmir, part of the union territory (Jammu & Kashmir) in India lies in the lap of the Himalayas. The people living in the far-flung areas of the region are largely dependent on the flora [22]. Forest resources are a source of income, employment, lodging, shelter, food, fodder, fuel, timber, vegetable, and medicine [20]. The local population belongs to different ethnic groups like Gujjar, Bakarwal, Pahari, and Kashmiri and possesses a unique wealth of information. Traditional knowledge has, however, been impacted by migration, urbanization, employment trends, and rising living standards. Our research followed key goals in order to document the loss of knowledge in the area: 1) to document the ethnomedicinal and cultural knowledge of local flora, 2) to analyze the cross-cultural use of the flora in the region, and 3) finally to identify the key indicator species utilized by each ethnic group using multivariate statistical analysis. By providing an answer to the aforementioned question, we will be able to provide additional cross-cultural ethnobotanical information on the forest resource that can support the preservation of regional plant diversity, culture, and their interactions.

## Research and methodology

### Study area

**Fig. 1:**
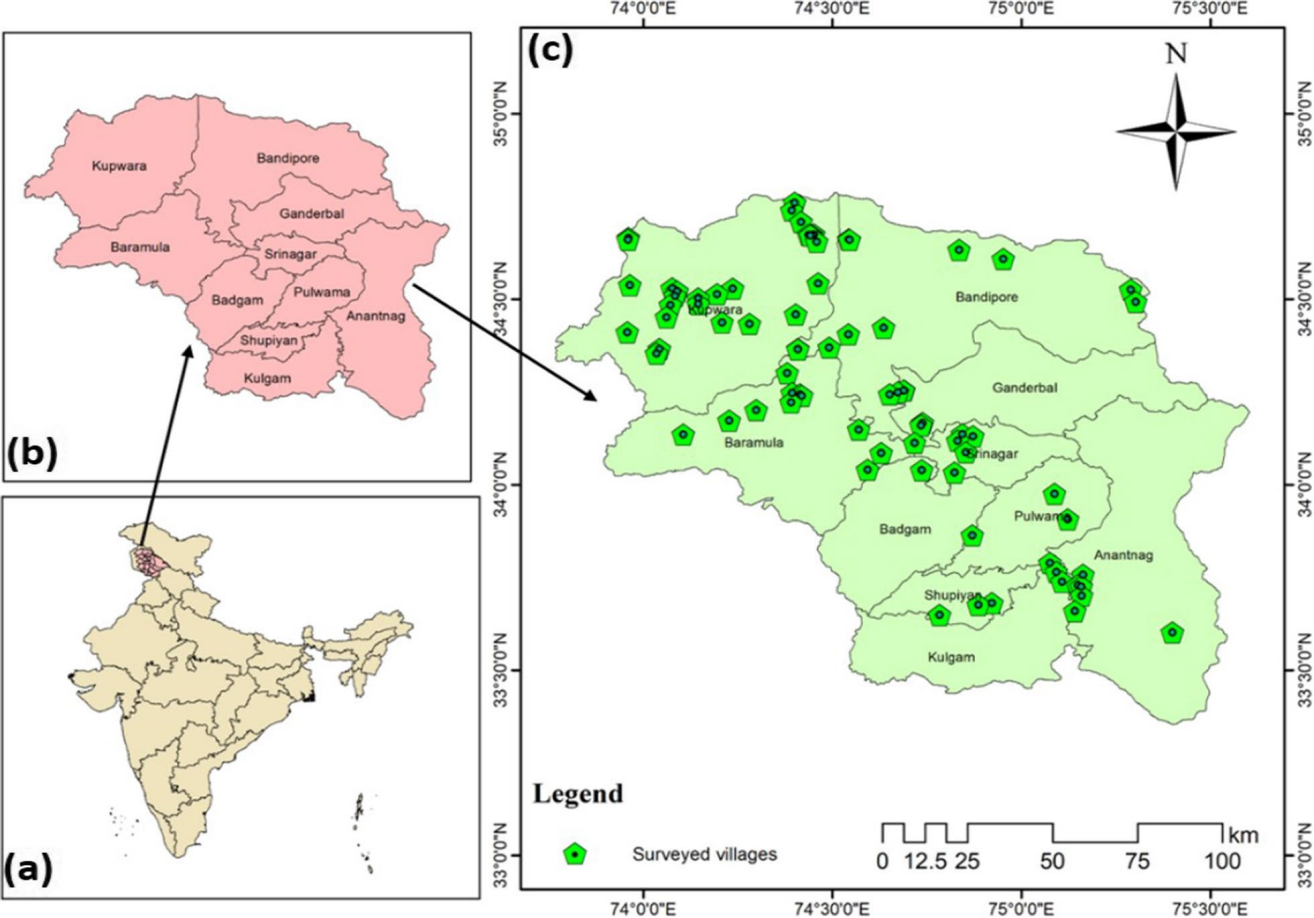
Map of the study area (**a**) India (**b**) Kashmir (**c**) showing the surveyed villages in the Kashmir valley, India

Kashmir division is part of the North-Western Himalayas and is currently administered as a part of the Union Territory (Jammu & Kashmir) in India, with 10 administrative districts (http://kashmirdivision.nic.in) (Fig. 1).

The region is mainly mountainous with maximum temperatures of 35 Cº in summer and -6 Cº in winter. The month of March receives the highest rainfall. The region is characterized by Himalayan dry temperate to subalpine forest types [14]. A rich cultural diversity with prominent ethnic communities like Pahari, Kashmiri and Bakarwal and Gujjar exists [23]. The languages spoken are Pahari, Kashmiri, and Gujjari, respectively. The local ethnic communities follow different religions, like, Islam, Hinduism, and Sikhism.

### Socioeconomic background

People living in the region are mainly engaged in agriculture (Pahari, Kashmiri) and cattle rearing (Bakarwal and Gujjar) [24]. Participants of the study also held Government jobs (6.09%), were wage laborers (14.63%), craftsmen (18.29%), herders (21.95%), housewives (9.75%), and shopkeepers (14.63%). In our study most of the traditional knowledge holders were old-aged people (56–75 +) (50%) followed by middle age-group (27–55 yrs.) (1.70%) and young age-group (18–26 yrs.) 18.29%). The majority of the population lives in rural areas. People widely use plants for primary health care and some clans have legacy of this traditional information about plants to treat a variety of ailments, with remedies often administered by traditional medicinal practitioners, locally called Hakeems.

### Demography of informants

A total of 82 respondents were selected for interviews, comprising 63 men and 19 women due to cultural limitations (Table 1). Before documentation, frequent visits were carried out in the study to ensure the cooperation of local people. The interviews followed [20, 25]. Prior to each interview, verbal prior informed consent was obtained, and the ISE Code of Ethics was followed (International Society of Ethnobiology, Code of Ethics. 2006) (https://www.ethnobiology.net). A translator was used to conduct the interviews in the respective native languages. As stipulated under the Nagoya Protocol, based on the agreement with the local participants, the ethnicity of the participants and the language information are not revealed. Using semi-structured questionnaires, we conducted interviews with individuals of various ages, gender, and occupational groups [10, 20]. Participants were asked about species, including information about the local name, parts used, disorders (ailments) treated, formulations, methods/techniques used in the preparation, adverse effects (if any), and use for treating that particular diseases. Most of the people in the study area had formal education (54.87%) and we found that illiterate people had less ethnomedicinal knowledge (45.12%). All the informants in our study followed Islam. At each research site, at least one knowledgeable informant assisted in specimen verification and herbarium preparation. Both photos and live plants were shown to the participants for identification and to obtain local names. The collected specimens were cross-checked with the assistance of taxonomist at the University of Kashmir, Srinagar (J&K), where the specimens were also deposited. To authenticate plant names, we used POWO 2019 (http://www.plantsoftheworldonline.org/).

**Table 1:** Demographic status of the respondents from the study area

Demographic features	Total percentage	Gujjar	Kashmiri	Pahari	Bakarwal
Respondents	82	24 (29.27%)	19 (23.17%)	21 (25.61%)	18 (21.96%)
Male	63 (76.82%)	17	15	14	16
Female	19 (23.17%)	7	2	6	4
Original language		Gojeri Urdu	Kashmiri Urdu	Pahadi Urdu	Gojeri Urdu
Religion		Shia and Sunni Islam Hinduism	Shia and Sunni Islam	Sunni Islam Hinduism	Sunni Islam
Nativity		Resident	Resident	Resident	Migratory
Marriages		Exogamous with other Muslims (Bakarwal)	Exogamous with other Muslims (Pahari) endogamous (Sikh)	Exogamous with other Muslims (Kashmiri, Gujjar) endogamous (Hindu)	Exogamous with other Muslims (Gujjar)
Livelihood source		Horticulture & cattle rearing	Horticulture pastoralism	Agriculture & cattle rearing	Pastoralism

### Ethnic communities

A total of four different ethnic communities were reported from the Kashmir division, i.e., Gujjar, Kashmiri, Pahari, and Bakarwal (Table 1). The Kashmiri ethnic group is the most dominant ethnic group in the study area, inhabiting the plain areas of the Kashmir valley; most of them followed Islam. All participants were using traditional medicine, although due to urbanization they were now largely consuming generic medicine. They have their own tradition and different language compared to other ethnic groups in the study area. Gujjar and Pahari ethnic groups live together and inhabit especially areas close to forested areas of the study area and are the second and third dominant ethnic groups in the study area. They share the same cultural tradition and mostly inter-marry, with a small difference in their languages. The Bakarwal ethnic group mostly migrate to the study area in spring from the Jammu region of Jammu & Kashmir and return in fall, using the alpine regions of the Kashmir valley. They have their own tradition and culture and there is a little similarity between Bakarwal and Kashmiri ethnic groups as they have little interaction between these two ethnic groups.

### Data analysis

The data were processed and analyzed in Microsoft Excel. Overall trends in fidelity level (FL) and use value (UV) were expressed through linear regression models through GraphPad Prism version 9 (GraphPad Software, CA, USA). We used fidelity level (FL) to determine which species were the most popular among residents in a given area [26]. To examine cross-cultural relationships of species utilization among ethnic groups a Venn diagram was created using Bioinformatics and Evolutionary Genomics software [16]. The indicator species for different ethnic groups were calculated based on the percentage of citations using the PAST software (version 10.3). Following the determination of the indicator values for each plant species, a linear regression model through the use of OriginPro version 9.8 was used to examine the correlation between the indicator values and the plant species used by different ethnic groups, including the Gujjar, Kashmiri, Pahari, and Bakarwal. The Jaccard Index (JI) was computed I to compare our data to previously published data from neighboring areas following González-Tejero et al. [27] using the given formula:

$${\text{JI}} = c * {1}00 \, / \, (a + b)\, - \,c$$

A qualitative comparison was made with 20 studies conducted in other Himalayan region by Ahmad et al. [28], Abbas et al. [29], Amjad et al. [30], Bano et al. [31], Bhat et al. [32, 33], Bhatia et al. [34], Farooq et al. [35], Khan et al. [36], Kumar et al. [37], Mir et al. [38], Ojha et al. [39], Rana et al. [40], Rashid et al. [41], Shah et al. [42], Sharma et al. [43], Singh et al. [44–46], and Wali et al. [47].

## Results and discussion

### Plant composition and distribution patterns

We recorded 46 species belonging to 25 different families used by the local people from four ethnic groups (Gujjar, Bakarwal, Pahari, and Kashmiri) of Kashmir valley (Table 2). The number of plant species identified in the research area was comparable to that found in past ethnobotanical investigations conducted in other Himalayan regions. For example, Barreda et al. [48] reported 53 plants from Monpa tribe in Eastern Himalayas; Mir et al. [9] reported 32 pants species from different ethnic groups of Kupwara, Kashmir Himalayas; Asif et al. [22] reported 29 species from various ethnic groups from remote tehsil (Karnah); Sher et al. [49] reported 53 plants from District Swat, Pakistan.

**Table 2:** List of plant species, local name, part used; preparation, form, mode of use, traditional cultural use across the four ethnic groups from the Western Himalayas

Scientific name/Abbreviation/Family/Voucher number	Local name	Part used	Mode of use	Preparation	Form	Diseases treated/ Local name (ethnic group)	Traditional cultural use	Ethnic groups Citation (indicator value)				LA	C	UV
								**G**	**K**	**P**	**B**			
*Aconitum chasmanthum* Stapf ex Holmes Acon.cha/ Ranunculaceae/SH-16	Patrees, Patis	Rhizome	Topically	Sun-dried rhizome is powdered and mixed with lukewarm water to make a paste	Paste, Raw	Abdominal infection/ Teda dard (G,B,P) Yed doud (K), Toothache/Dand doud (K) Dand nal dard (G)	–	8 (0.718)	6 (0.439)	10 (0.521)	12 (0.323)	×	36	0.44
*Aconitum heterophyllum* Wall. ex Royle/Acon.het/Ranunculaceae/ SH-05	Pewak, Patris	Leaves	Orally	Leaves are dried are used raw	As whole	Stomach problem/ Yed doud (K)	Leaves used as vegetables	25 (0.044)	25 (0.042)	8 (0.393)	6 (0.571)	×	62	0.86
		Rhizome	Orally	Rhizome is dried in the sun and then turned into a powder to be consumed with water	Powder	Indigestion/ Badhazmi (G,P,B) Piles/ Bawaseri (K)								
*Aconitum violaceum* Jacquem. ex Stapf./Acon.vio/Ranunculaceae/SH-11	Mohand, DudhiAtees	Fruit	Orally	Fruits are powdered after being dried and consumed with water	Powder	Intestinal infection/ Andramn doud (K), Asthma/ Asthma (K,P,B)	Leaves are used as vegetables	5 (0.808)	6 (0.429)	9 (0.601)	11 (0.413)	×	31	0.38
*Androsace rotundifolia* Hardw./Andr.rot/ Primulaceae/SH-23	Uzm posh, Uzm	Rhizome	Orally	After being dried in the shade and ground into a powder, rhizomes are consumed with water	Powder	Eye inflammation/ Ankh suje di (G, P, B) Aech doud (K)	–	4 (0.891)	5 (0.451)	6 (0.725)	6 (0.606)	×	21	0.26
*Angelica glauca* Edgew./Ange.gla/Apiaceae/SH-18	Sapsade, Chora	Rhizome	Orally	Rhizomes are taken with water after being shade-dried and ground to a powder	Powder	Epilepsy/ Jala (G, P) Stomach cramps/ Peechi (K) Badhazmi (P,B)	Rhizome used for eradication of rodents	8 (0.893)	4 (0.373)	10 (0.516)	11 (0.414)	×	33	0.40
*Aquilegia nivalis* (Baker) Falc. ex B.D. Jacks./Aqui.niv/Ranunculaceae/ SH-13	Zoaneil, Columbine	Rhizome	Topically	Squeezed raw rhizome produces a fine liquid	Liquid	Foot inflammation/ Pau suj de (G,P,B)	–	8 (0.717)	__	06 (0.719)	16 (0.151)	×	30	0.37
*Arisaema jacquemontii* Blume/Aris.jac/Araceae/SH-38	Hapatmakai, Hapetgogej	Fruit	Topically	Fruits are powdered and shade-dried, then combined with lime juice to make a paste	Paste	Leprosy/ Daney pad gayeah (G, B)	–	17 (0.218)	__	__	20 (0.131)	√	37	0.45
		Seeds	Orally	Dryed seeds are ground into a paste and combined with honey	Paste	Bronchitis/ Fefda vich dard (B)								
*Arnebia benthamii* (Wall. ex G. Don) I.M. Johnst./Arne.ben/Boraginaceae/SH-32	Khazaban, Gawzaban	Leaves	Orally	To obtain liquid, freshly picked leaves are squeezed	Liquid	High fever/Taap (G, P), Taaf (K) Abdominal pain/ Teda daed (B, P) Mead doud (K)	Tea is obtained from flowers	22 (0.126)	23 (0.066)	2 (0.826)	5 (0.625)	×	53	0.65
		Flowers	Orally	To obtain liquid, flowers are squeezed	Liquid	Brain tonic/ Epilepsy Jala di (G, P), Cold/ Sardi (B, P) Zukam (K)								
*Atropa acuminata* Royle ex Lindl./Atro.cum/Solanaceae/SH-02	Mait brand/Yabrooj	Rhizome	Orally	Rhizome is ground into a powder and shade dried before being consumed on an empty stomach	Powder	Neuro-tonic/ Dimag khrab (G,P,K)	–	9 (0.649)	8 (0.066)	11 (0.448)	__	×	28	0.34
*Bergenia ciliata* (Haw.) Sternb	Zakhmihayat, Bud mawe	Leaves, Rhizome	Topically	Rhizome and leaves are ground into a paste and combined with water	Paste	Injuries/ Zakhim (G, P, B)	Leaves used as lid for utensils. Rhizome is used for herbal tea	23 (0.092)	__	22 (0.373)	7 (0.481)	×	42	0.51
*Bunium persicum* (Boiss.) B. Fedtsch./Buni.per/Apiaceae/SH-09	Zeeur, Kalazeera	Seeds	Orally	After drying, seeds are powdered	Powder	Indigestion/ Badhazmi (G,K,P)	Seeds are used as spice	4 (0.886)	18 (0.128)	3 (0.804)	__	×	25	0.31
*Corydalis cashmeriana* Royle/Cory.cas/Papaveraceae/SH-35	Haz posh, Mast kul	Rhizome	Orally	Dry, powdered rhizome is consumed with water	Powder	Liver disease/ Kalaji di dard (G, P, B)	–	9 (0.365)	__	12 (0.432)	11 (0.412)	×	32	0.39
*Corydalis govianiana* Wall./Cory.gov/Papaveraceae/SH- 44	Sang e-harb, Bhutya	Rhizome	Topically	Dried, powdered rhizome is combined with water	Powder	Hair loss/ Bal nikaldey (P, G) Mas narun (K) Lice infestation in animals/ Jue (P, B) Zuw dawa (K)	–	14 (0.365)	6 (0.432)	4 (0.778)	7 (0.565)	×	31	0.38
*Delphinium cashmerianum* Royle/Delp.cas/Ranunculaceae/SH-07	Mori, Noori	Leaves	Orally	The dried and powdered leaves are consumed with lukewarm water	Powder	Paralysis/ Waj (G, K) Harkat (B,P)	–	13 (0.411)	3 (0.499)	8 (0.625)	7 (0.569)	×	31	0.38
			Orally	Dryed leaves are ground up and combined with water to create paste	Powder	Heart tonic/ Dil dawa (K)								
*Dipsacu sinermis* Wall./Dips.ine/Caprifoliaceae/SH-01	Wapalhakh, Wopalhakh	Leaves	Orally	To get liquid, fresh leaves are squeezed	Liquid	Body inflammation/ Sujan (G, B, P) Internal injuries/ Zakhim (P, B) Constipations/ Marood (G,P)	Fodder	9 (0.658)	__	16 (0.205)	7 (0.567)	×	32	0.39
*Dolomiaea costus* (Falc.) Kasana& A.K. Pandey/Dolo.cos/Asteraceae/SH-10	Kuth, Kooth	Leaves	Orally	Powdered leaves are ground from shade-dried leaves and taken with lukewarm water	Powder	Arthritis/ Harkat (G, P, B) Reh (K)	Leaves are used as vegetables	28 (0.041)	9 (0.325)	7 (0.657)	6 (0.608)	×	47	0.57
		Rhizome		Sun-dried rhizome is powdered and consumed with lukewarm water	Powder									
*Dolomiaea macrocephala* DC. ex Royle/Dolo.mac/Asteraceae/SH-26	Dope, Gugal	Rhizome	Orally	Rhizome is sun-dried, ground up, and consumed with lukewarm water	Powder	Neurological disorders/ Makax doud (K)	Roots are used as vegetables	6 (0.757)	3 (0.503)	4 (0.777)	23 (0.062)	×	36	0.44
		Root	Orally	Sun-dried roots are ground into a powder and taken with lukewarm water	Powder	Fever/ Taap (G, B, P) Taaf (K), Back pain/ Lakh vich dard (G, P) Kamras doud (K)								
*Euphorbia wallichii* Hook. f./Euph.wal/Euphorbiaceae/SH-30	Gudsochal, Kaaliheerbi	Stem Latex	–	–	Latex	Warts/ Poori (G, P) Skin diseases/ Khushe (G)	–	10 (0.561)	__	15 (0.289)	__	√	25	0.30
*Ferula jaeschkeana* Vatke/Feru.jae/Apiaceae/SH-03	Heng, Hingpatri	Root	Orally	Sun-dried roots were ground into a powder and mixed with lukewarm water	Powder	Snake bite/ Saaf ka dasna (P) Gastric problem/ Ted dard (G, P)	–	16 (0.265)	__	13 (0.396)	__	×	29	0.35
*Fragaria nubicola* (Hoof. f) L./Frag.nub/Rosaceae/SH-08	Yangtaesh, Briij	Fruit	Orally	In order to obtain a liquid, raw fruits are squeezed	Liquid	Neurological disorders/ Demag khrab (K)	Fruit are used as food, and fodder	11 (0.524)	17 (0.173)	9 (0.606)	__	×	37	0.33
*Fritillaria cirrhosa* D. Don/Frit.cir/Liliaceae/SH-22	Sheethkhar, Kakoli	Rhizome	Orally	Rhizome is taken with lukewarm water after being sun-dried, stored for months, and ground to powder	Powder	Tuberculosis/ Tee-Bee (G, P, K) Abdominal pain/ Yed doud (K)	–	8 (0.721)	9 (0.338)	25 (0.043)	10 (0.431)	×	52	0.63
*Fritillaria imperialis* L./Frit.imp/Liliaceae/SH-20	Banud, Hapat posh	Bulb, Flower	Topically	Flowers and bulbs are combined, dried, then ground with water and applied	Powder	Frost bite/ Sho (P, K)	Flowers are used for decoration	__	17 (0.178)	13 (0.39)	__	√	30	0.37
*Gentiana kurroo* Royle/Gent.kur/Gentianaceae/SH-31	Neelibuti, Nilkanth	Leaves	Topically	To make paste, fresh leaves are ground	Paste	Skin allergies/ Alergy (G, K)	–	13 (0.414)	15 (0.258)	__	__	×	28	0.34
			Orally	The powdered leaves are taken with water after being shade-dried	Powder	Liver problem/ Kalaji vich dard (G) Khranmaz khrab (K) Gastric pain/ Maid doud (K)								
*Geranium wallichianum* D. Don ex Sweet/Gera.wal/Geraniaceae/SH-37	Ratanjote, Laljar	Rhizome	Orally	Rhizomes are sun-dried, ground, and consumed with water	Powder	Inflammation of gall bladder/ Tilpita vich sujan (G, P) Arthritis/ Harkat (B)	Rhizomes are ground to obtain sap, used as dye	12 (0.437)		23 (0.066)	14 (0.303)	×	49	0.60
*Inula racemose* Hook. f./Inul.rac/Asteraceae/SH-33	Maleen, Chakiphool	Rhizome	Orally	Powdered, shade-dried rhizomes are consumed with water.	Powder	Dysentery/ Marood (G) Nose bleeding/ Nak nal loo (P)	–	14 (0.361)	__	15 (0.289)	__	×	29	0.35
*Iris hookeriana* Foster/Iris.hoo/Iridaceae/SH-46	Mazarmund, Mazamond	Rhizome	Orally	Rhizomes are sun-dried, ground, and consumed with water	Powder	Rheumatism/ Reh pay gaye (P, B)	Roots are used as rodenticide	__	__	10 (5.211)	21 (2.141)	×	31	0.38
*Jurinea dolomiaea* Boiss./Juri.dol/Asteraceae/SH-42	Duphe, Guggal	Roots	Orally	The powdered roots are taken in the morning with water	Powder	Diabetes/ Sugar (G, B, P)	Raw roots are used for tooth cleaning	24 (0.064)	__	9 (0.617)	15 (0.212)	×	48	0.59
*Lagotis cashmeriana* Rupr./Lago.cas/Scrophulariaceae/SH-06	Chilkaur, Tragbol	Rhizome	Orally	To obtain liquid, fresh rhizome is crushed and squeezed	Liquid	Dyspepsia/ Teda vich jalan (G, P, B)	–	7 (0.742)	__	9 (0.617)	15 (0.215)	×	31	0.38
*Morina longifolia* Wall. ex DC./Mori.lon/Caprifoliaceae/SH-15	Kandiyari/kim	Leaves	Orally	The leaves are powdered after being dried and consumed with lemon water	Powder	Neurological disorders/ Dimag vich dard (G, P, B) Anthelminthic/ Malap (G,P,B)	–	5 (0.807)	__	6 (0.712)	14 (0.297)	×	25	0.30
*Phytolacca acinosa* Roxb./Phyt.aci/Phytolaccaceae/SH-39	Hapatfal, Rechkaguch	Roots	Topically	Powdered dried roots are used	Powder	Pus in feet/ Poo (G, P)	–	11 (0.527)	__	15 (0.279)	__	√	26	0.32
*Picrorhiza kurroa* Royle ex Benth./Picr.kur/Polygonaceae/SH-12	Koud, Kutki	Leaves	Orally	Sun-dried leaves are ground into a powder and consumed with lukewarm water	Powder	Intestinal infection/ Anthadi vich dard (P, B)	–	__	__	15 (0.281)	14 (0.294)	×	29	0.35
*Podophyllum hexandrum* Royle/Podo.hex/Berberidaceae/SH-19	Wanwangun, Khakdi	Leaves, Fruits	Topically	Fresh leaves are ground into a paste and combined with lemon drops	Paste	Warts/ Poori (G, B, P) Heart disease/ Dil dard (P)	Fruits are also used as food	18 (0.215)	__	18 (0.143)	15 (0.217)	√	33	0.40
*Potentilla nepalensis* Hook./Pote.nep/Rosaceae/SH-17	Chai kul, Chaekulll	Rhizome	Orally	To obtain liquid, fresh rhizome is ground and squeezed	Liquid	Neurological disorders/ Dimag dard (G, P, B)	–	11 (0.524)	__	14 (0.307)	11 (0.412)	×	36	0.44
*Primula denticulata* Sm./Prim.den/Primulaceae/SH- 25	Childer, wan posh	Roots	Orally	Roots are dried powdered and taken with fresh spring water	Powder	Urinary infection/ Muth nal kharbi (G, P)	–	14 (0.364)	__	17 (0.173)	__	×	31	0.38
*Prunella vulgaris* L./Prun.vul/Lamiaceae/SH-21	Kalyuth, kalyuth	Flower	Topically	Fresh flowers are ground to obtain paste	Paste	Migraine/ Mygran (K) Cold and fever/ Taff te teer (K), Foot fever/ Khuran taaf (K) Stomach gas Madis gas (K)	__	__	35 (0.023)	__	__	×	35	0.43
*Rheum spiciforme* Royle/Rheu.spi/Polygonaceae/SH-29	Pamichari	Leaves	Topically	Leaves are dried, ground to obtain powder	Powder	Injuries/ Zakhmi (P, B)	Leaves are used as vegetables	__	__	25 (0.044)	24 (0.046)	×	49	0.60
		Rhizome	Orally	Fresh rhizome is ground and squeezed to obtain liquid	Liquid	Leprosy/ Dand pey gayeah (P) CarbuncleDaney (B)								
*Rheum webbianum* Royle/Rheu.web/Polygonaceae/SH-24	Pumbchalan, Pamichari	Leaves	Orally	The powdered leaves are taken with spring water after being dried and ground	Powder	Rheumatism/ Reh (G,B,P,K)	–	4 (0.892)	20 (0.112)	20 (0.107)	8 (0.466)	×	52	0.63
*Rhododendron campanulatum* D. Don/Rhod.cam/Ericaceae/SH-40	Inga, Ingae	Bark	–	Fresh bark is used	Raw	Tooth pain/ Dand dard (G,P,B)	Stems used as fuel	9 (0.653)	__	5 (0.74)	24 (0.049)		38	0.46
			Orally	Sun-dried bark is ground up and powdered	Powder	Drug addiction								
*Saussurea simpsoniana* (Fielding & Gardner) Lipsch./Saus.sim/Asteraceae/SH-14	Jogibadshah, Jogipadshah	Leaves, Flower	Orally	The dried, powdered leaves and flowers are both taken with milk	Powder	Infertility/ Mardana Kamzoori (G,B)	Flowers presented to anybody are a wish for good luck	10 (0.563)	__	__	22 (0.089)	×	32	0.39
*Swertia chirata* Buch.-Ham. ex Wall/Swer.chi/Gentianaceae/SH-28	Chirayata, Chirayeta	Roots	Orally	The roots are powdered and taken with lukewarm water after being dried	Powder	High fever/ Taap (G, P)	–	15 (0.302)	__	13 (0.394)	__	×	28	0.34
*Thymus linearis* Benth/Thym.lin/Lamiaceae/SH-34	Jayeen, Junglijayen	Seeds	Orally	Dried and powdered seeds are consumed along with lukewarm water	Powder	Fever/ Taaf (K) Abdominal pain/ Teda dard (G, P) Mead doud (K)	Seeds are used as spice	19 (0.149)	15 (0.258)	__	14 (0.434)	×	48	0.59
		Leaves	Orally	To extract liquid, young leaves are squeezed	Liquid	Leg pain/ Zang doud (K)								
*Trigonella emodi* Benth./Trig.emo/Leguminosae/SH-41	Junglimeeth, Boti	Leaves		The powdered, dried leaves are consumed with goat milk	Powder	Body weakness/ Kamzoori (P, G)	–	11 (0.528)	__	18 (0.151)	__	×	29	0.35
*Trillium govanianum* Wall. ex D. Don/Tril.gov/Melanthiaceae/SH-27	Tulhakh, Tripater	Leaves	Orally	To obtain liquid, freshly picked leaves are squeezed	Liquid	Leg pain/ Zang doud (K) Tang pach gaye (G, P)	Leaves used as vegetables	22 (0.149)	12 (0.274)	12 (0.434)	__	×	46	0.56
		Rhizome		Rhizome is sun-dried, powdered, and consumed with curd	` Powder									
*Valeriana jatamansi* Jones/Vale.jat/Caprifoliaceae/SH-36	Musk bala, Khasi	Leaves	Orally	To get liquid, fresh leaves are squeezed	Liquid	Abdominal pain/ Teda dard (G) Mead doud (K) Fever/ Taaf (K)	–	18 (0.217)	16 (0.227)	__	__	×	34	0.41
*Verbascum thapsus* L./Verb.tha/Scrophulariaceae/SH-43	Wan tamook, Janglitamook	Seeds, Stem	Orally, Topically	The seeds are powdered, dried, and consumed with warm water	Powder, Paste	Chronic respiratory diseases/ Sush khrab (K) Skin burns Saad gaya (P) Dazun (K)	–	16 (0.257)	20 (0.104)	__	__	×	36	0.44
*Viburnum grandiflorum* Wall. ex DC/Vibu.gra/Adoxaceae/SH-45	Kulmosh/Kuch/SH- 35	Leaves	Orally	Fresh leaves are used	Raw	Muscle sprain/ Maas pach gaya (G) Maaz fatun (K)	Ripe fruits eaten as food and branches as fuel wood. And leaves as fodder	15 (0.293)	16 (0.214)	__	__	×	31	0.38
		Fruits	Orally	Ripe fruits are used	Raw	Spinal cord strain/ Reck-adig doud (K)								

G = Gujjar, P = Pahari; K = Kashmiri; B = Bakarwal; Uses shown in bold = novel; LA = Lethal attribution; ×  = Non-lethal, √ = Lethal; C = citation; UV = Use value

**Fig. 2:**
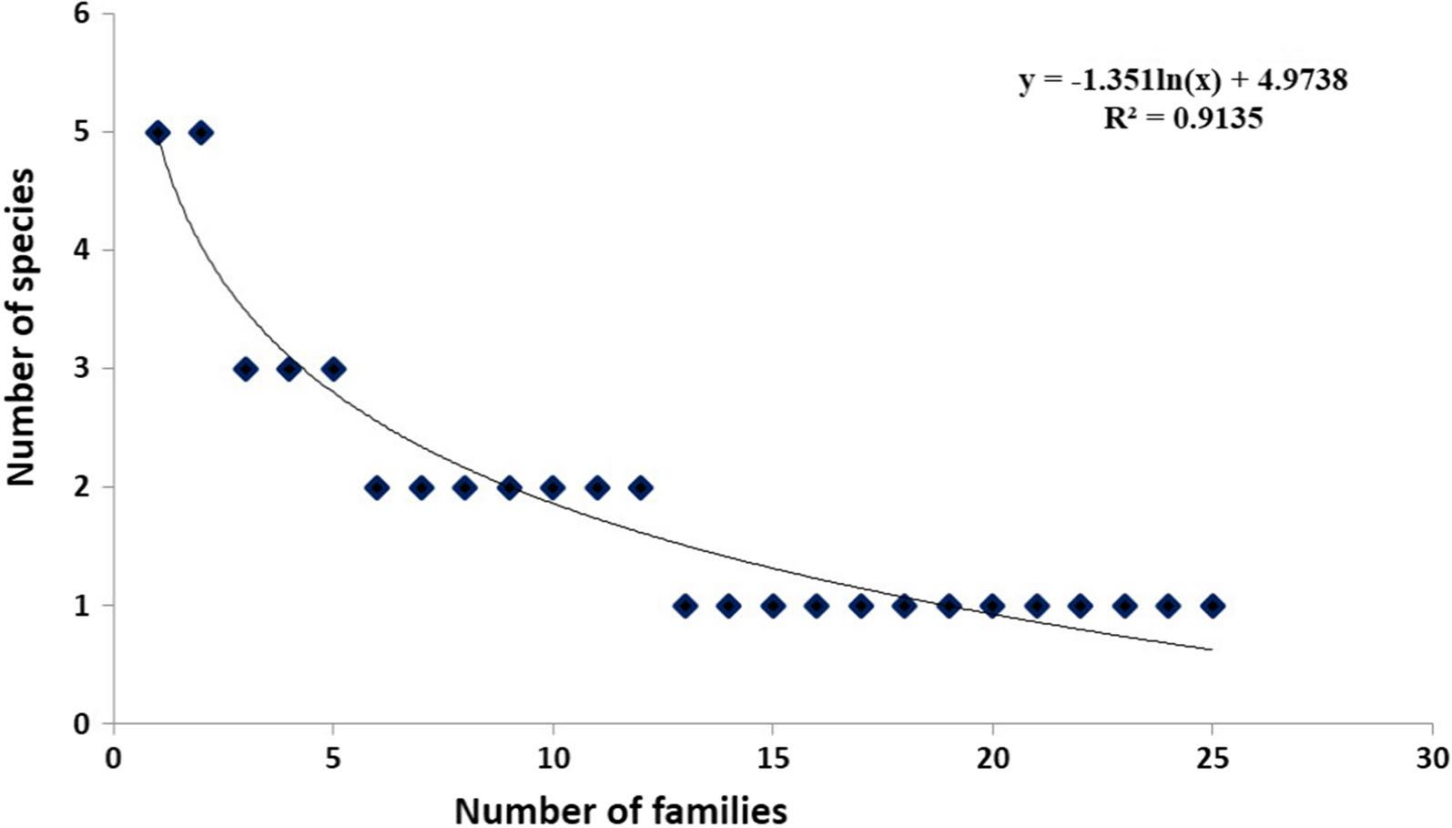
Species family relationship of the vegetation in the high-altitude Western Himalaya

The distribution of species across 25 families was disproportionate, with 7 families (Asteraceae, Ranunculaceae, Apiaceae, Caprifoliaceae, Polygonaceae, Gentianaceae, and Lamiaceae) accounting for about half of the species and 18 families accounting for the other half, including 13 families represented by single species. The species family relationship is shown in Fig. 2. The dominant families were Asteraceae and Ranunculaceae (11% each) followed by Caprifoliaceae (7%) (Table 2). Asteraceae was found dominant in many biomes, primarily in open habitat ecosystems [50]. Several studies have concluded that the Asteraceae was also the most important or useful family in the surrounding areas of Pakistan and Kashmir Himalayas [9, 51, 52]. Likewise, [53] also reported Asteraceae as a dominant family in the Highlands of Gasa District, Bhutan. Ranunculaceae was the second leading family in our study area. Kayani et al. [54] also reported Ranunculaceae as a leading family from the high-altitude of Pakistan. Because of their alkaloids, sterols, flavonoids, and glycosides, plants in the Asteraceae and Ranunculaceae are known as a rich source of medicinal products used to treat a variety of ailments [55, 56]. Furthermore, the current study discovered that families had unequal distribution patterns, with 13 monotypic families, it agrees with earlier ethnobiological research from other Himalayan areas [20, 22].

### Traditional cultural use categories

The most common use of plants was for medicine (72%) followed by food (14%) and fodder (5%) (Table 2). This suggests that the high-altitude plant resources are significant in all facets of life for those who reside in remote areas, especially in terms of providing for their fundamental needs in terms of food, shelter, livelihoods and healthcare. Several other studies also reported similar results from other Himalayan regions like [20] from District Reasi, Northwestern Himalaya, [57] from Kashmir Himalayas [14], and from high-altitude Trans Himalaya. People often prefer to use traditional medicine because it is widely available locally, less expensive, has few perceived side effects, its accessibility, and simplicity in administration, and there is a growing importance of medicinal plants commonly used in folk medicine [58]. However, many species may fail to pass clinical testing due to their toxicity and biocompatibility issues.

### Parts used

**Fig. 3:**
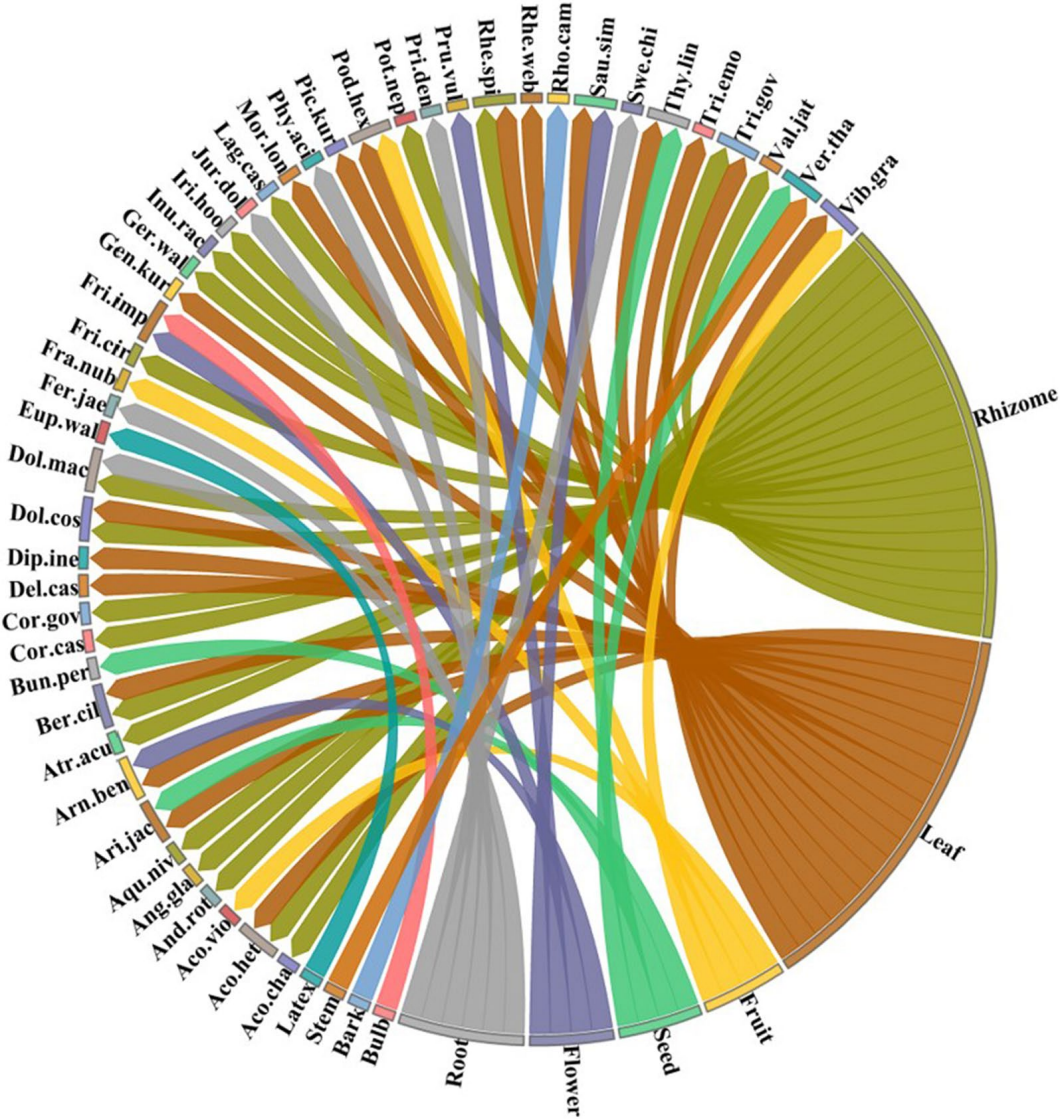
Distribution of plant species in various plant parts categories used in the region. The direction of the lines depicts which plant species is linked with which part used and thickness of each bar indicates the degree of parts used in each category

Different parts of plants were used with a significant difference (*χ*2 = 90.587, df = 7, *p* < 0.001). Rhizomes were most utilized (45% of uses), followed by leaves (31%) and fruits, flowers, and seeds (5% each) (Fig. 3). Rhizomes are widely used in pharmaceutical preparations due to their high concentration of bioactive components [11]. Similar findings were reported by various ethnobotanists including [9] from Kashmir Himalayas, [59] from Northern Ethiopia, and [2] from tribal communities in the Western Himalaya. Excessive use of rhizomes, particularly in the case of threatened species, should be avoided because it can have a detrimental impact on population and growth, as well as lead to extinction [9]. Leaves might have the potential to be a source of valuable drugs in addition to food. The presence of alkaloids in them explains why they are often employed as a remedy in traditional medicine so efficiently [14].

### Form of remedy preparation

In the current study, powder (59%) was the most frequent preparation form, followed by paste (15%) and decoction (14%) (Table 2). Rokaya et al. [60] also reported powder form as the common form used by local inhabitants of Nepal India. The patients regarded powdered preparations as quite effective, and it was utilized whenever possible. Similarly, findings were reported by [61] from Himalayan India.

### Diseases cured

**Fig. 4:**
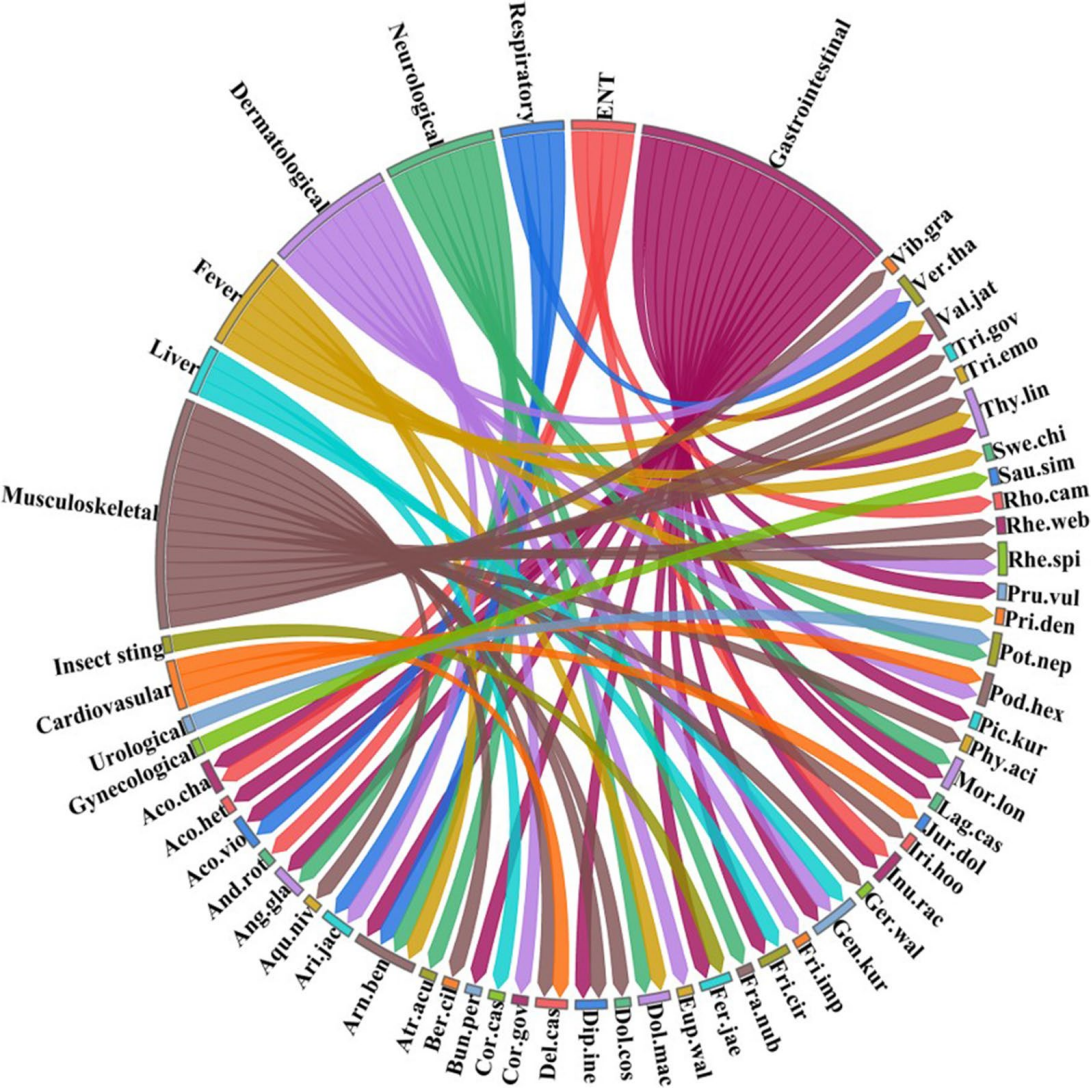
Distribution of plant species in various disease treated categories used in the region. The direction of the lines depicts which plant species is linked with which disease and thickness of each bar indicates the degree of plants used to treated disease in each category

A total of 33 aliments were treated with plants, and most species (25%) were employed for gastrointestinal disorders, followed by musculoskeletal diseases (20%) and dermatological problems (12%) (Fig. 4, Table 2). The possible reason behind these results might be that gastrointestinal disorders are common in these areas due to a lack of hygienic conditions, malnutrition, and a lack of pure water. Similar results were reported by [62] and [12] from Pakistan; [63] from Northern Nigeria; and [9, 35] and [30] from various ethnic groups of Northern Himalaya. Among the uses of plants for medicine, the treatment of gastrointestinal diseases holds a significant role in many regions [64–67].

### Novelty of the study

This study's list of 46 wild plants was cross-checked against 20 published articles from the entire Himalayan region (Table 3). This comparison helped to reveal variances of local wild plants that existed across various populations and places as shown in the Jaccard Index (JI). In the current study, the JI values ranged from 10.87 to 0.64 (Table 3). The highest value was reported from District Kupwara [33; 10.87] followed by the Sindh forest, Jammu & Kashmir, India [36; 9.40], and Kedarnath Wildlife Sanctuary, Himalaya, India [32; 7.03]. Due to similar geographic or climatic conditions, the greater JI score demonstrates the similarity in vegetation types between the two places. The minimum JI was calculated in the study of Bandipora District, Kashmir Himalaya, [44; 0.64], where only 1 similar plant was reported with the current site because there is a significant difference in the geography; the reported site is a mountainous region with low altitude, whereas the current study is a high-altitude mountain region.

**Table 3:** Jaccard index value for plants

Study area	Region	Year	NT	NTSU	NTDU	NTCBS	TRAA	TRSA	PTSU	PTDU	JI	Citation
Neelum Valley	AJK, Pakistan	2017	50	2	3	5	45	41	4.00	6.00	5.49	[28]
Baltistan region	Karakorum range-Pakistan	2017	84	1	2	3	81	43	1.19	2.38	2.36	[29]
ToliPeerNational Park	AJK, Pakistan	2017	121	3	4	7	114	39	2.48	3.31	4.38	[30]
Skardu Valley	Karakoram-Himalayan, Pakistan	2014	50	0	4	4	46	42	0.00	8.00	4.35	[31]
Kedarnath Wildlife Sanctuary	Himalaya, India	2013	152	3	10	13	139	33	1.97	6.58	7.03	[32]
Kupwara District	Jammu & Kashmir, India	2021	107	3	12	15	92	31	2.80	11.21	10.87	[33]
Udhampur	Jammu & Kashmir, India	2014	166	0	2	2	164	44	0.00	1.20	0.95	[34]
Dhirkot	AJK, Pakistan	2019	140	1	3	4	136	42	0.71	2.14	2.20	[35]
Sindh Forest	Jammu & Kashmir, India	2022	82	4	7	11	71	35	4.88	8.54	9.40	[36]
Shankaracharya Hill	Jammu & Kashmir, India	2015	130	0	2	2	128	44	0.00	1.54	1.15	[37]
Northern Jammu and Kashmir	Jammu & Kashmir, India	2022	109	1	4	5	104	41	0.92	3.67	3.33	[38]
Bageshwar district	Central Himalaya, India	2020	70	1	3	4	66	42	1.43	4.29	3.57	[39]
Chamba district	Western Himalaya, India	2019	83	2	3	5	78	41	2.41	3.61	4.03	[40]
Azad Jammu and Kashmir	AJK, Pakistan	2018	73	1	3	4	69	42	1.37	4.11	3.48	[41]
Poonch districts	Jammu & Kashmir, India	2015	104	2	4	6	98	40	1.92	3.85	4.17	[42]
Uttarakhand	Sub-Himalayan, India	2013	24	0	1	1	23	45	0.00	4.17	1.45	[43]
Bandipora District	Kashmir Himalaya, India	2016	111	0	1	1	110	45	0.00	0.90	0.64	[44]
Rudraprayag district	Western Himalaya, India	2017	78	2	2	4	74	42	2.56	2.56	3.33	[45]
Jasrota Hill	Western Himalaya, India	2020	121	0	2	2	119	44	0.00	1.65	1.21	[20]
Diamir district	Western Himalayas, Pakistan	2022	61	0	2	2	59	44	0.00	3.28	1.90	[47]

*NT* Number of taxa, *NTSU* Number of taxa with similar uses, *NTDU* Number of taxa with dissimilar uses, *NTCBS* Number of taxa common in both studies, *TRAA* Taxa reported in allied area, *TRSA* Taxa reported in study area, *PTSU* Percentage of taxa with similar uses, *PTDU* Percentage of taxa with dissimilar uses, *JI* Jaccard index

**Fig. 5:**
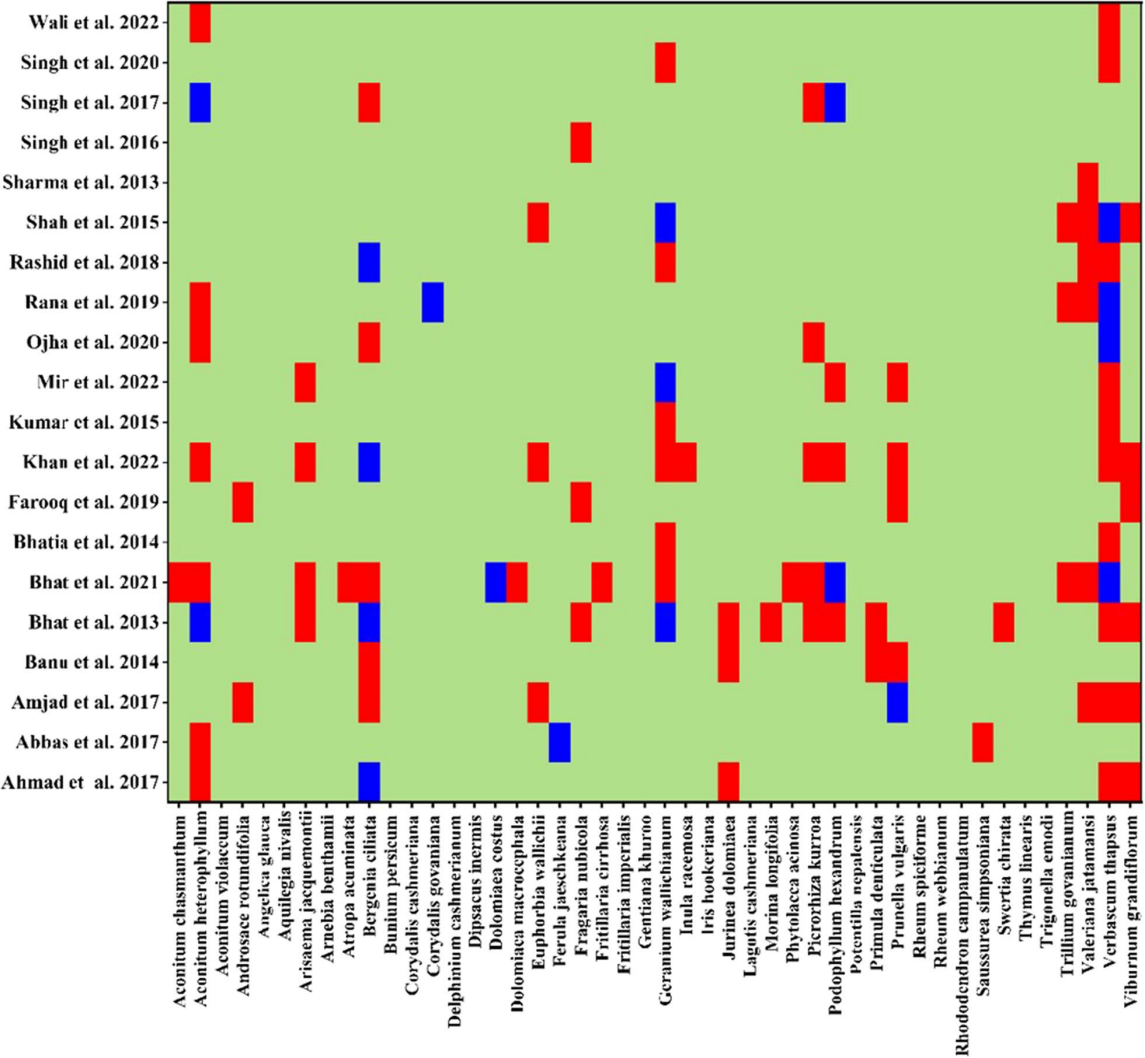
Comparison of the current study with previous studies in the Himalayan region. Red color showed the common plant in both study with dissimilar use, while blue color shows the common plants in both study with similar use

The current study also reports 18 wild species that have rarely been documented for treating human ailments in the ethnobotanical literature: *Aconitum violaceum, Angelica glauca, Aquilegia nivalis*, *Arnebia benthamii, Bunium persicum*, *Corydalis cashmeriana*, *Delphinium cashmerianum*, *Dipsacus inermis, Fritillaria imperialis, Gentiana kurroo*, *Iris hookeriana*, *Lagotis cashmeriana*, *Potentilla nepalensis*, *Rheum spiciforme, Rheum webbianum, Rhododendron campanulatum, Thymus linearis*, and *Trigonella emodi.* While 28 medicinal plants had already been reported in the available literature, we documented novel utilization for 18 of these species (Fig. 5). The present study reported new uses for 24% plant species, e.g., rhizome of *Corydalis govianiana* for killing lice in animals, raw roots of *Jurinea dolomiaea* for tooth cleaning, seeds of *Verbascum thapsus* for respiratory diseases, leaves of *Bergenia ciliata* as lid for utensils, flowers of *Saussurea simpsoniana* were presented to anybody a wish for good luck, leaves of *Dipsacus inermis* were used for internal injuries and body inflammation, leaves of *Arnebia benthamii* as brain tonic, leaves of *Viburnum grandiflorum* against muscle sprain, leaves of *Aconitum violaceum* used as food, rhizome of *Geranium wallichianum* as dye, bark of *Rhododendron campanulatum* to overcome drug addiction, rhizome of *Aconitum heterophyllum* for stomach problems and piles.

### Use value

**Fig. 6:**
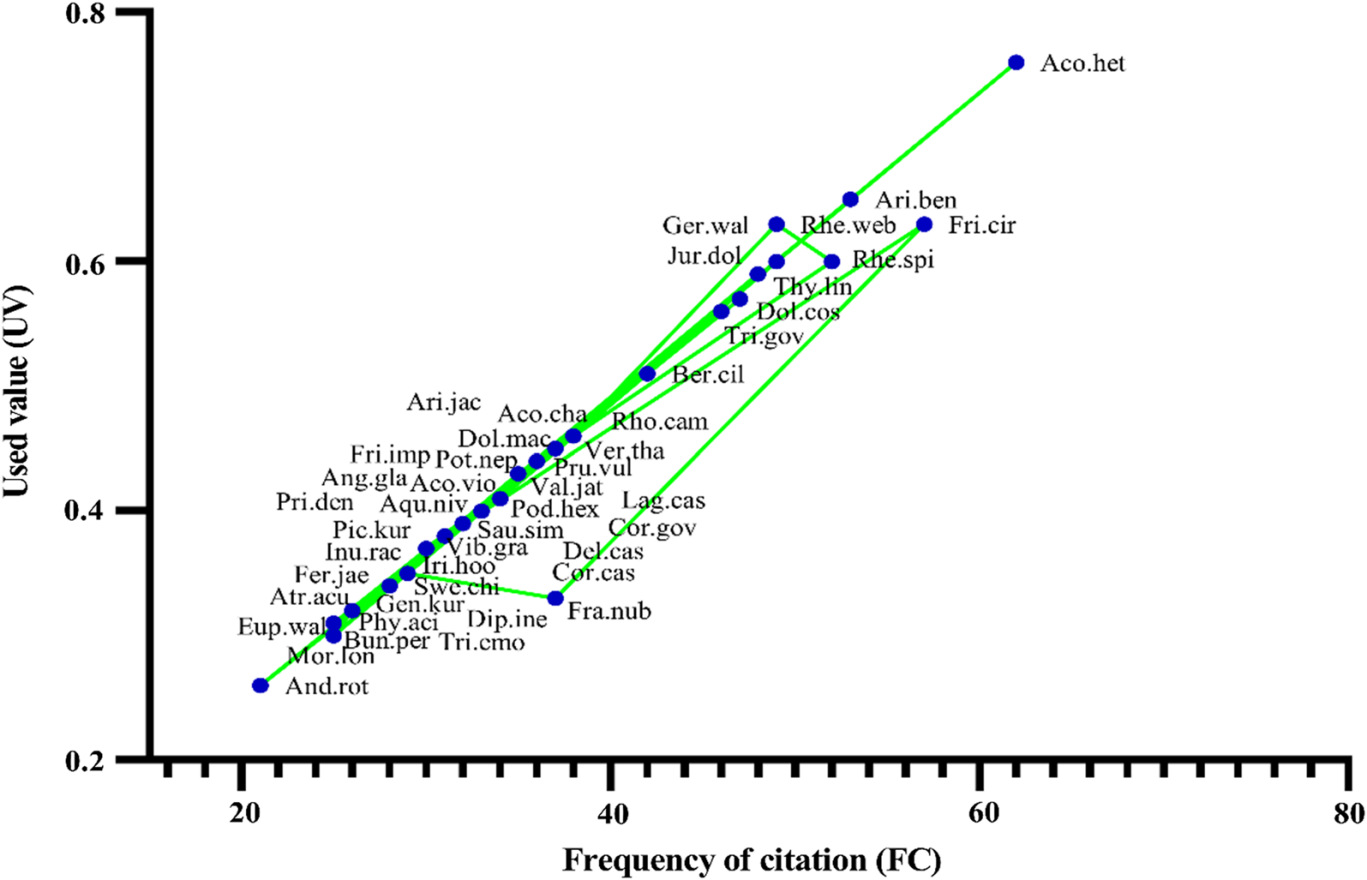
Relationship between use value (UV) and frequency of citation (FC). The full plant names are presented in Table 2

In the present study, the highest UV of 0.86 was calculated for *Aconitum heterophyllum* and the lowest UV of 0.26 for *Androsace rotundifolia* (Fig. 6; Table 2). Due to their widespread distribution and widespread knowledge of therapeutic applications among the local population, medicinal plants in the research location had high UV levels [35, 68, 69].

### Cross-cultural analysis

**Fig. 7:**
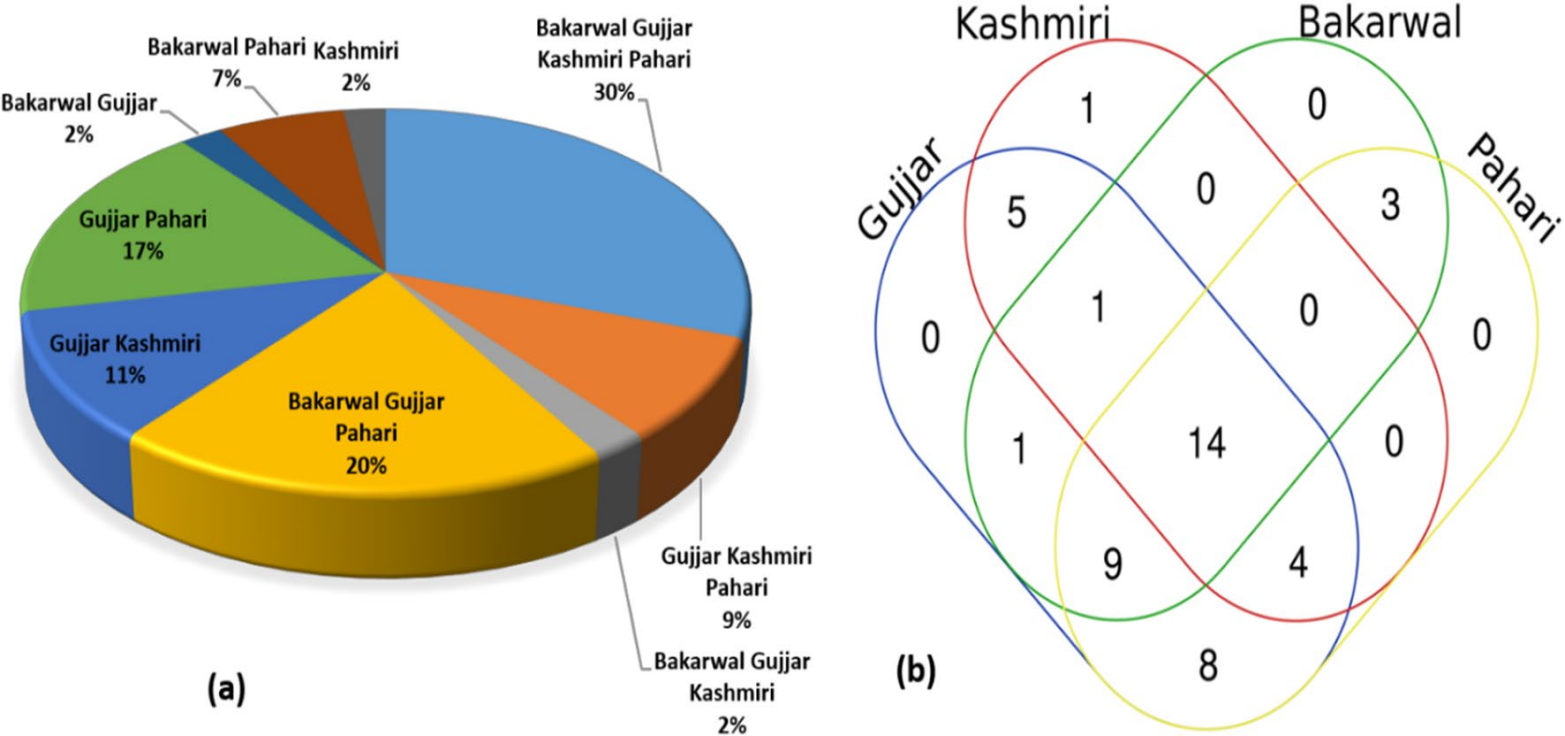
**a** Percentage of similarity ** b** Venn diagram showing pattern of ethno-veterinary usage pattern of plant resources in different ethnic groups of Kashmir region

We examined how social, economic, and cultural factors influenced plant resource utilization patterns among various ethnic groups in the region. Across cultural relationships, the Gujjar and Pahari showed greater similarity (17%), whereas the least overlap (2%) was observed between Bakarwal, Gujjar, and Kashmiri (Fig. 7a).

The reason for the greater similarity of Gujjar and Pahari lies in the fact that both ethnic groups share a common geographical landscape, while both are exogamous to each other (Table 1), whereas the Bakarwal, Gujjar, and Kashmiri have different cultural identities, live in different areas of the region, and not surprisingly showed the least relationship. The cross-cultural analysis revealed that all ethnic groups used 14 species on a regular basis followed by nine species shared by the Bakarwal, Gujjar, and Pahari and minimum one species (*Saussurea simpsoniana*) by Bakarwal, Gujjar, and Kashmiri (Fig. 7b). Meanwhile, Kashmiri was the only ethnic groups with idiosyncratic species (*N* = 1) *Prunella vulgaris*, which may be accounted for by the fact that the aforementioned species thrives in habitats near rivers, streams, and canals, which are located at relatively elevations, as well as by the fact that the aforementioned ethnic group use it commonly during COVID-19 to treat cold, fever, and migraine. Many other researchers conducted similar cross-cultural analyses, revealing the overlapping and uniqueness of species due to cultural, landscape, availability, and socioeconomic factors such as [14] from the Trans Himalayas; [25] and [70] from Pakistan Himalaya; [10] and [71] from Kashmir Himalaya; and [72] from Eastern Morocco and Eastern Andalusia.

**Fig. 8:**
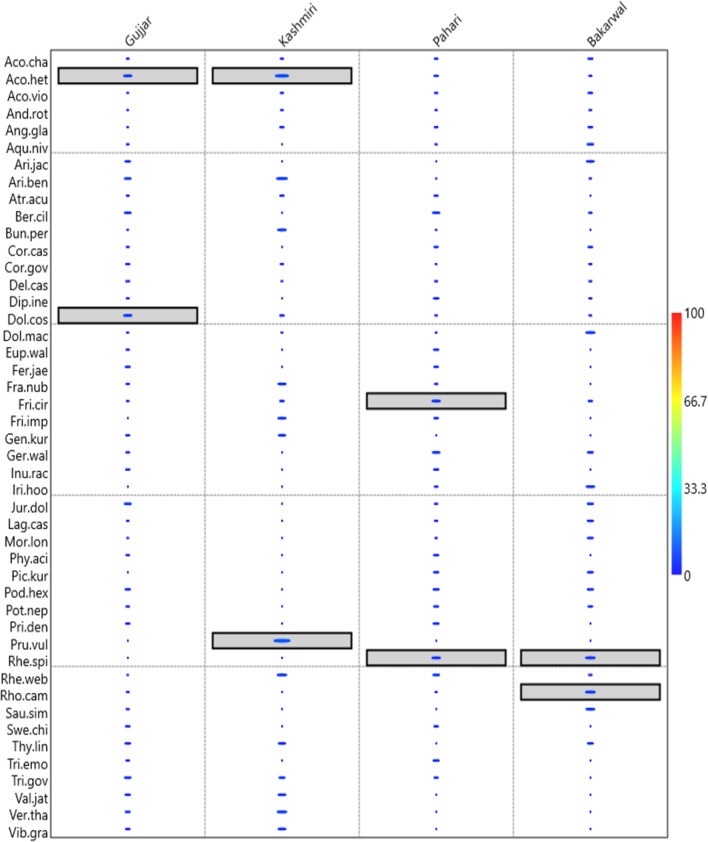
Indicator values of species in different ethnic groups in different ethnic groups of Kashmir region. Indicator plants were highlighted in box in different groups

The Venn diagram can, however, not provide a clear picture of plant usage; for example, if a specific ethnic group uses N plants, it does not specify whether it is used by one informant or by maximum members of that ethnic group. We used indicator species analysis for the first time to solve this problem. The analysis of indicator species revealed a clear distinction between key species in four ethnic groups (Fig. 8). In the Gujjar ethnic group, *Aconitum heterophyllum* and *Dolomiaea costus* had significant indicator value, the reason behind this being that these plants grow in abundance near forests and are easily available, and also have a wide range of uses and also support livelihood, while *Rhododendron campanulatum* and *Rheum spiciforme* all had significant p values in the Bakarwal ethnic group, because this ethnic group spends the majority of their time in high-altitude pastures, making them frequently use these plant species for medicine, food and fuelwood. *Aconitum heterophyllum* and *Prunella vulgaris* were indicator species of the Kashmiri ethnic group, both these species are having the multiple medicine usage (particularly in COVID-19). In the Pahari ethnic group, *Fritillaria cirrhosa* and *Rheum spiciforme* were indicator species; all of these species had numerous medicinal uses in addition source of livelihood. The majority of species with high indicator values were associated with particular ethnic communities and heavily consumed by them. *Aconitum heterophyllum* was a common indicator species between Gujjar and Kashmiri ethnic groups, while *Rheum spiciforme* was a common indicator species in Pahari and Bakarwal ethnic groups (Fig. 8). The reason was that these plants were frequently used in traditional medicine, and Gujjar and Bakarwal communities primarily extracted them from the surrounding forest area and selling them to the Kashmiri community, which primarily resides in the lower reaches of the Kashmir valley. In this way, information on medicinal usage and means of support are passed from one community to another.

Some of the commonly used plant species used by all four ethnic groups were *Aconitum heterophyllum, Angelica glauca*, *Fritillaria cirrhosa, Rheum webbianum, Dolomiaea costus,* and *Arnebia benthamii.* The reason behind this was that these species were all medicinal and were used in various forms as well as having cultural and traditional values. The ethnomedicinal knowledge of these species was held mostly in the Gujjar, Bakarwal, and Pahari ethnic groups. The Kashmiri ethnic group had less ethnomedicinal knowledge because of the preference for allopathic medicine. People often had to walk by foot about 10–15 km to reach the areas where medicinal plants were collected.

Medicinal plants used by Gujjar and Pahari ethnic groups were *Ferula jaeschkeana, Euphorbia wallichii, Primula denticulata* and *Phytolacca acinosa.* The reason behind this great similarity between these two ethnic groups lies in culture, language, and tradition. These ethnic groups live near forests and visit higher altitudes in summer along with their livestock. There were least plant species which used by Bakarwal and Kashmiri ethnic group, given their very limited interactions between these two ethnic groups and their different culture and tradition as well as language. The Gujjar and Pahari acted as knowledge-transferring agents between Bakarwal and Kashmiri ethnic groups. The highest number of plant species was used by Gujjar, Pahari, and Bakarwal ethnic groups. The interaction between these ethnic groups usually occurred in summers in the alpine regions of the study area where all these ethnic groups live together along with their livestock.

### Relationship between indicator values and the number of plants used by different ethnic groups

**Fig. 9:**
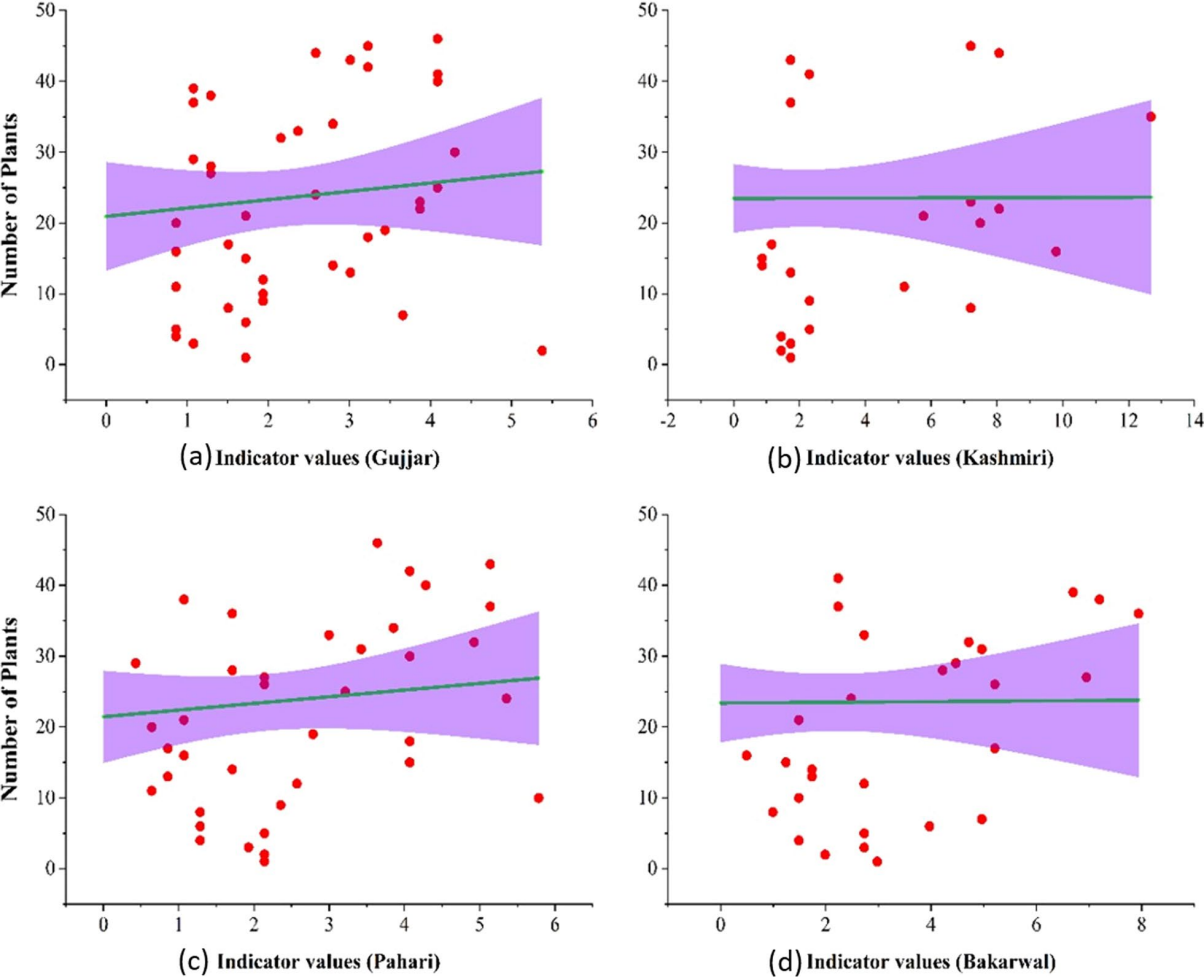
Linear regression between plants and indicator values of plant used by different ethnic groups **a** (Gujjar) **b** Kashmiri **c** Pahari **d** Bakarwal from the study area

The results showed that the indicator values and the number of plants had a linear relationship (i.e., positive correlation) with the Gujjar (*R*^2^ = 0.013, intercept = 20.94, slope = 1.17), Kashmiri (*R*^2^ = 1.08, intercept = 23.47, slope = 0.013) and Pahari (*R*^2^ = 0.014, intercept = 21.45, slope = 0.94), while the relationship between the number of plants represented a negative correlation with indicator values of the Bakarwal ethnic group (*R*^2^ = − 0.022, intercept = 23.39, slope = − 0.049) (Fig. 9).

Our data enabled an investigation into the links between the indicator values of numerous widely used plants with the relative importance of ethnobotanically used species as well as various ethnic groups. The positive connection indicates cultural preferences for certain plant use and underlines the cultural significance of each species. Such positive combinations create the door for their future applications. The highest R^2^ value for the community showed strong relationships with indicator values of medicinal flora.

### Plants and livelihoods

The ethnic people living in the Himalayas have a close relation with the local flora [22]. We recorded 16 plant species (*Trillium govanianum*, *Arnebia benthamii*, *Aconitum heterophyllum*, *Aconitum chasmanthum*, *Dolomiaea costus*, *Fritillaria cirrhosa*, *Jurinea dolomiaea*, *Saussurea simpsoniana*, *Rheum webbianum*, *Podophyllum hexandrum*, *Gentiana kurroo*, *Angelica glauca*, *Dolomiaea macrocephala*, *Bunium persicum, Rheum spiciforme*, and *Picrorhiza kurroa*) being sold by ethnic communities to improve their income, and the traditional health practitioners (Hakeems) also used these species for making medicinal preparations. The ethnic groups with the highest plant use were Bakarwals and Gujjar. Due to strict faith in traditional medicine, the demand of the plant species was very high, hence people earned handsome incomes. If trade of plant species continues at the same rate, it is possible that in various species will vanish from the region the near future, ultimately affecting the biodiversity of the region. In the past, due to high market value, the unchecked exploitation of different species resulted already in the loss of biodiversity and a threat to many species [73, 74].

### Plant toxicity

In the present study, local people indicated that *Rhododendron campanulatum*, *Podophyllum hexandrum*, *Arisaema jacquemontii*, *Fritillaria imperialis*, *Euphorbia wallichii* and *Phytolacca acinosa*, besides having medicinal value, did also possess poisonous potential if harvested in the inappropriate stage. Plants with lethal attribution constituted 8.69% of the total documented species.

Different plant parts were reported as responsible for toxicity. For instance, unripe fruits of *Podophyllum hexandrum*, *Arisaema jacquemontii*, *Phytolacca acinosa*, early-stage leaves of *Rhododendron campanulatum*, immature bulb of *Fritillaria imperialis*, latex from rhizomes of *Euphorbia wallichii*. The lethality of the plant/part can often be ascribed to alkaloids present [11, 75]. It is important to note that local people suffered losses in the form of livestock/human deaths as a result of the consumption of these plant species. As a result, our study can be used as a written reference for a safer future utilization of these plant resources.

## Conclusions

The current study will help to convince policymakers to concentrate on ethnic groups' social sustainability in order to achieve long-term sustainable resource management. We focused on documenting the eroding traditional knowledge across the cultural use of the flora in the region, observing that ethnic groups sharing a geographical environment and being exogamous with one another showed the highest overlap in the use of plant resources, whereas those with distinct cultural identities, living in separate parts of the territory showed the least similarity. The Gujjar and Pahari acted as knowledge-transferring agents between Bakarwal and Kashmiri ethnic groups. We found that key indicator species, *Aconitum heterophyllum, Rhododendron campanulatum, Fritillaria cirrhosa, Rheum spiciforme, Dolomiaea costus,* and *Prunella vulgaris,* were connected to particular ethnic communities and were all used for food, medicine, and other essential purposes. This study may help future generations preserve their traditional knowledge in writing and advance the creation of scientifically sound protection plans for their cultural and botanical resources.

## Data Availability

All data have already been included in the manuscript.
